# The structural and functional determinants of the Axin and Dishevelled DIX domains

**DOI:** 10.1186/1472-6807-9-70

**Published:** 2009-11-12

**Authors:** Matthias T Ehebauer, Alfonso Martinez Arias

**Affiliations:** 1 European Molecular Biology Laboratory, c/o DESY, Building 25a, Notkestraße 85, 22603 Hamburg, Germany; 2 Department of Genetics, University of Cambridge, Downing Road, Cambridge, CB2 3EH, UK

## Abstract

**Background:**

The *dishevelled *and *axin *genes encode multi-domain proteins that play key roles in WNT signalling. Dishevelled prevents β-catenin degradation by interfering with the interaction of β-catenin with the degradation-mediating Axin-APC-GSK3β complex. This interference leads to an accumulation of cytoplasmic β-catenin, which enters the nucleus and interacts with transcription factors that induce expression of Wnt-target genes. Axin, as a component of the degradation-mediating complex, is a potent negative regulator of Wnt signalling, whereas Dishevelled is a potent activator. Both Dishevelled and Axin possess a DIX (Dishevelled/Axin) domain, which mediates protein-protein interactions, specifically homodimerization.

**Results:**

An evolutionary trace analysis of DIX domains identified conserved residues which, when mapped onto the crystal structure of the Axin DIX domain and a comparative model of the Dishevelled DIX domain, allow their categorization as residues of either structural or functional importance. We identify residues that are structural and functional determinants of the DIX domain fold, as well as those that are specific to homodimerization of Axin and Dishevelled.

**Conclusion:**

This report provides the first explanation of the mutant phenotypes caused by non-synonymous substitutions in the Dishevelled and Axin DIX domain by correlating their presumed functional significance with molecular structure.

## Background

Signalling by members of the Wnt family of secreted glycoproteins relies on dynamic interactions between protein complexes to regulate transcription, the cytoskeleton and cell adhesion [1,2]. Binding of Wnt proteins to their receptors, members of the Frizzled and LRP families, leads to the activation of Dishevelled, a multi-domain protein that mediates many functions of Wnt [1-3] and this triggers a sequence of protein-protein interactions, which culminate in specific effector activities.

In the 'canonical' pathway, Wnt proteins regulate transcription of specific genes by modulating the amount, activity and intracellular location of β-catenin, a multi-domain protein with a role in linking Cadherins to the cytoskeleton, in addition to being an effector of Wnt signalling [4]. In the absence of Wnt, a soluble pool of β-catenin is targeted for degradation by a multiprotein complex assembled around the scaffolding protein Axin and in which glycogen synthase kinase 3β (GSK3β) is the catalytic component. GSK3β phosphorylates β-catenin, thus targeting it for degradation by the proteosome [1,4]. Upon Wnt signalling, Dishevelled prevents β-catenin degradation by interfering with the interaction of the Axin-APC-GSK3β complex and β-catenin [1,5-8]. This activity involves a direct interaction between Dishevelled and Axin [8] and leads to an accumulation of cytoplasmic β-catenin, which enters the nucleus and interacts with transcription factors of the LEF/TCF family, inducing the transcription of Wnt-target genes.

The central role of Dishevelled in Wnt signalling and the key role of Axin in the activity of β-catenin suggest that the interaction between these two proteins represents a central event for Wnt activity. The interaction between these two proteins is mediated for the most part through their DIX (Dishevelled/Axin) domains, although other regions of both proteins are also involved [5,6,8]. A third protein containing a DIX domain, and also involved in Wnt signalling, is the coiled-coil protein DIX-domain-containing 1 (DIXdc1, also known as Ccd1). It acts as a positive regulator of WNT signalling and although the molecular mechanism underlying this function is not clear [9,10], it interacts with Dishevelled through its DIX domain. In contrast, the interaction of DIXdc1 with Axin is not dependent on its DIX domain [10].

**Figure 1:**
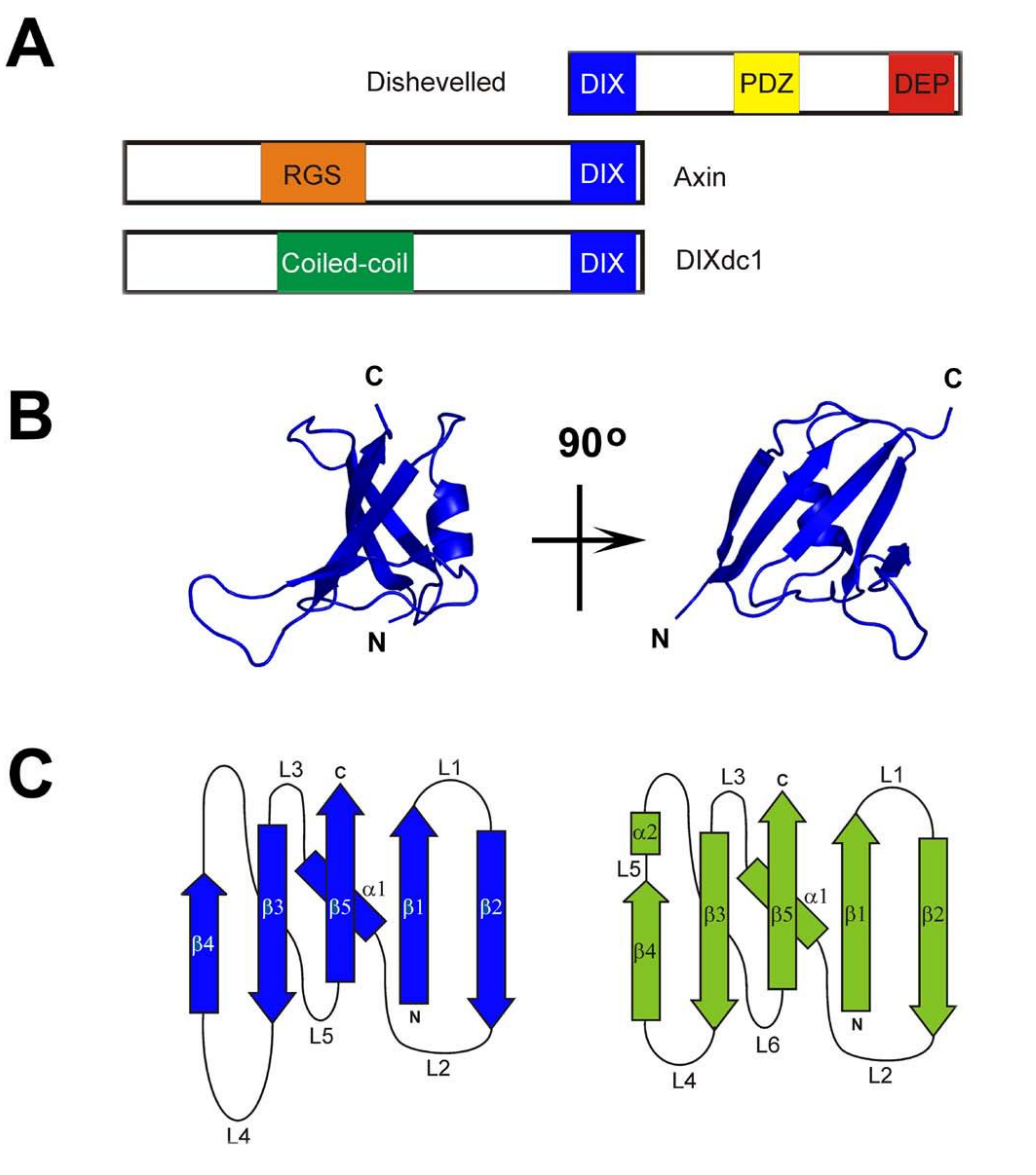
**DIX domain structure**. (A) Architecture of the DIX domain containing proteins Dishevelled, Axin and DIXdc1. Dishevelled has a N-terminal DIX domain, a PDZ and DEP domain. Axin has a RSG domain and C-terminal DIX domain. DIXdc1 has a coiled-coil domain and a C-terminal DIX domain. (B) The β-grasp fold structure of the Axin DIX domain, a β-sheet against which is packed a α-helix (1WSP). (C) Topology of the Axin DIX domain (blue) and ubiquitin (green). The ubiquitin-like β-grasp fold of both proteins consists of a 5-stranded β-sheet in which the strands are arranged in a 2-1-5-3-4 order starting at the protein N-terminus. In ubiquitin an additional helix, helix α2, connects strand β4 with strand β5. In Axin this helix is absent.

The DIX domain is a region of approximately 83 to 85 residues in length located at the N-terminus of Dishevelled and at the C-terminus of Axin and DIXdc1 (Figure 1A). While the domain architecture in Figure 1A are those of human Dishevelled 1, rat Axin 1 and human DIXdc1, the relative location of the domains in each of these protein's homologues is similar. The DIX domain has been shown to be involved in dimer formation, which has led to the suggestion that this kind of interactions might mediate the regulation of the activity of the Axin-based complex [5,11-13]. A crystal structure of the Axin DIX domain (1WSP) has recently been reported [14]. It consists of a five-stranded β-sheet in which the β-strands are in the order 2-1-5-3-4 and a single α-helix (residues T776 to L784) that packs into the concave groove of the β-sheet (Figure 1B). This fold is similar to the common β-grasp fold of the ubiquitin superfamily [15] and is widespread among proteins (Figure 1C). Proteins containing ubiquitin-like folds often have an additional helix between β-strands 4 and 5, which is absent form the DIX domain. Examples of such proteins are ubiquitin itself and the Phox-Bem1 (PB1) domain-containing proteins whose PB1 domains form asymmetric dimers [16-18] that are similar to those of the Axin DIX domain [14].

Only a limited number of functionally important amino acids in DIX domains have been identified and additional single or multiple substitutions may shed light on the precise function of this domain. Here we have performed a sequence-structure analysis of the DIX domain of Axin and Dishevelled using the evolutionary trace analysis approach first described by Lichtarge et al. [19], to identify residues that could be selectively targeted in mutagenesis studies. Trace residues were mapped onto the crystal structure of the Axin DIX domain and onto a comparative model of the Dishevelled DIX domain, allowing their categorization as residues of either structural or functional importance. These residues could be further categorized as either conserved or class-specific. Most conserved residues cluster on the protein-protein interaction interface required for DIX homotypic interactions. Many of these are absolutely conserved, whereas the class-specific surface-exposed trace residues are likely required for specificity of DIX domain interactions. The analysis reported here provides a structure-based explanation of the mutant phenotypes caused by non-synonymous substitutions in the Dishevelled and Axin DIX domain.

## Methods

### Comparative modelling

A comparative model of the human Dishevelled 1 DIX domain, residues M1 to E85, was created using Modeller 9v1 [20] based on the coordinates of chain B residue C750 to D832 of the asymmetric unit of the Axin DIX domain (1WSP) [14]. The latter was identified as a structural homologue by the homology recognition algorithm FUGUE [21]. A structure-based sequence alignment of the Dishevelled and Axin DIX domain was created using ClustalW [22] and manually edited in order to ensure that secondary structural elements remained gap-free. Secondary structure for the human Dishevelled 1 DIX domain was predicted using JPred [23]. Model geometry was validated using PROCHECK [24]. Topologically similar folds to that of the Axin DIX domain were identified using DALI [25].

### Evolutionary trace analysis

One hundred and twenty sequence homologues of the human Dishevelled 1 DIX domain were identified using BLASTp with the database search parameter set to non-redundant protein sequences. Of these, 63 were retained for evolutionary trace analysis (see Additional file 1). Protein sequences that were discarded for this analysis were either redundant sequences, hypothetical proteins or sequences annotated as 'unknown' proteins. Of the retained sequences, 22 were of Axin, 31 of Dishevelled and 10 of DIXdc1. These sequences were aligned using ClustalW [22] and manually edited to ensure no gaps were inserted into areas of known secondary structure. Evolutionary trace analysis of the resulting sequence alignment was performed using TraceSuiteII [26] according to the method of Lichtarge et al. [19]. This method, in brief, assumes that architecture-defining residues are mostly invariant, whereas residues that are functionally important may undergo numerous substitutions over time. Furthermore, it assumes that proteins that have diverged recently would share greater sequence identity than those that diverged earlier. A phylogenetic tree is constructed base on a multiple-sequence alignment and this tree then arbitrarily partitioned (denoted by vertical lines across the tree). Each partition will contain a different set of tree nodes. The sequences associated with these nodes are aligned to generate a consensus sequence for that partition. Trace residues in a particular partition are those that are conserved. Residues conserved in the first few partitions (close to the root of the tree) were conserved in the distant past and are most often important for structural reasons. Residues conserved in later partitions (close to the branches of the tree) were conserved more recently and are associated with function. Trace residues are then mapped onto a molecular structure, which is examined visually to identify clusters of residues.

Trace residues were mapped onto the structure of the Axin DIX domain (1WSP) and the comparative model of the Dishevelled DIX domain. Based on their location in the relevant structure, the trace residues were classified as either (1) conserved and buried, (2) conserved and surface exposed, (3) class-specific and buried or (4) class-specific and surface exposed. Class-specific residues are residues that are conserved only in a subset of sequences, such a subset being defined as a class. Residues were classified as buried if less than 30% of their side chain is solvent accessible.

## Results and discussion

### Comparative modelling

A comparative model of the Dishevelled DIX domain was created in order to gain some insight into the functionally significant elements of this domain. We used the sequence of the human Dishevelled 1 DIX domain to search for structural homologues to act as templates for comparative modelling. The Axin DIX domain of *Rattus norvegicus *(1WSP) was the only structural homologue of the human Dishevelled DIX domain identified with certainty by the homology recognition server FUGUE (Z-score 19.18). The *R. norvegicus *Axin and the human Dishevelled DIX domain share 30% sequence identity. A sequence-based secondary structure prediction of the human Dishevelled DIX domain showed that the position and identity of secondary structural elements match those of the Axin DIX domain crystal structure (data not shown; [14]). Given the degree of sequence identity, which is an established measure of comparative model accuracy [20], and their similar predicted secondary structure content and related protein-protein interaction function, it is likely that the overall structures of the Dishevelled and the Axin domains are similar. The final model of the DIX domain of human Dishevelled 1 contained all of the protein's N-terminal 85 residues, 93.4% of which are in the most favoured region of the Ramachandran plot, 6.6% are in the additionally allowed regions. None are in the disallowed region.

### Structural homology

The DIX domain has a typical β-grasp fold characteristic of the ubiquitin superfamily. A search of the Protein Databank using the fold comparison server DALI identified PB1 domains as structurally similar to DIX domains, sharing similar topology and, after examination of their dimeric structures, similarity to the putative Axin DIX homodimer evident in the crystal structure of that domain [14]. The PB1 domains are known to form asymmetric homodimers and/or heterodimers using acidic residues on β-stands 3 and 4 of one domain that interact with basic residues on β-strands 1-2 of an adjacent domain [16-18]. These interactions are conserved to the extent that consensus sequences can be defined for different PB1 types [27]. These neither match with any sequence of the DIX domain, nor is the complementary of electrostatic surface potentials of PB1 domains evident in the Axin DIX crystal structure. The DIX domain may therefore represent another example of a β-grasp fold forming homotypic dimers, but through interactions that are not predominantly electrostatic in nature.

### Evolutionary trace analysis general results

**Figure 2:**
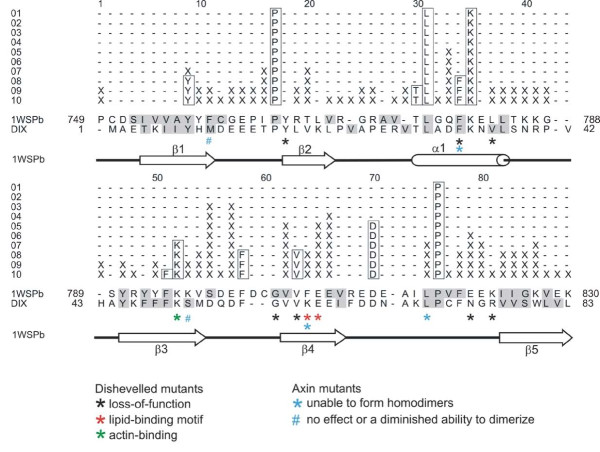
**Evolutionary trace analysis**. Traces of partitions P01 to P10 aligned with amino acid sequences of the Axin DIX domain structure (1WSPb = chain B of the asymmetric unit; residues 749-830) and the human Dishevelled DIX domain comparative model (DIX; residues 1-83). Conserved residues are boxed, class-specific residues are denoted by X, solvent-exposed side-chains are shaded grey. The secondary structure assignment of the Axin DIX domain is given below the trace. # coloured cyan are sites of non-synonymous amino acid substitution in Axin that have no effect on or only diminished ability to dimerize and * coloured cyan are unable to dimerize [12]. * coloured black are sites of non-synonymous amino acid substitution in Dishevelled that lead to a loss-of-function [29], whereas * coloured red are residues constituting the putative Dishevelled lipid-binding motif and * coloured green are required for Dishevelled actin-binding [28,30].

In order to determine the possible structural or functional role of specific residues in the DIX domain, sequences of DIX domain-containing proteins were aligned and the observed sequence conservation mapped onto the DIX domain structures. The alignment of available Axin, Dishevelled and DIXdc1 proteins shows that there are only few residues that are absolutely conserved (see Additional file 2). However, there are several residues that are conserved within each of the families and most of these are located on strand β4. The alignment in Additional file 2 was used to construct a dendrogram (see Additional file 3) and a trace of conservation (Figure 2). Analysis of the mapped traces for partitions 01 to 10 reveal several buried residues that form part of the domain's core, as well as a large cluster of surface exposed residues centred around the loop L4 connecting β-strands 3 and 4 (Figure 2 and Additional file 3). Since this analysis included DIX domain sequences from all three known families of DIX-domain containing proteins, absolutely conserved trace residues can be considered structural and functional determinants of the DIX fold. Traces were also derived for the set of 31 Dishevelled homologues on their own, with similar results to that of the larger group (data not shown). However, noise levels were significantly higher due to the greater sequence conservation among Dishevelled homologues. The most divergent Dishevelled sequence known, that of the sea squirt *Ciona intestinalis*, still shares 45% sequence identity with human Dishevelled. This is also evident in Additional file 3, where the divergence of vertebrate and most invertebrate Dishevelled family members is evident only much later (P06-P08) than the divergence of vertebrate and invertebrate members of the Axin family (P02).

The higher degree of sequence conservation observed for the DIX domain of Dishevelled may reflect the fact that it is associated with multiple functions: DIX domain homotypic interactions, its interaction with lipids and binding to actin [28], which might have necessitated the conservation of many residues over evolutionary time. The DIX domain of Axin has only one reported function, homotypic DIX-DIX protein interactions, which likely allowed a greater degree of divergence between homologues. Too few homologues of DIXdc1 have been reported to make a similar conclusion and the scarcity of sequence data for this family precluded its detailed analysis.

### Identification of trace residues

**Figure 3:**
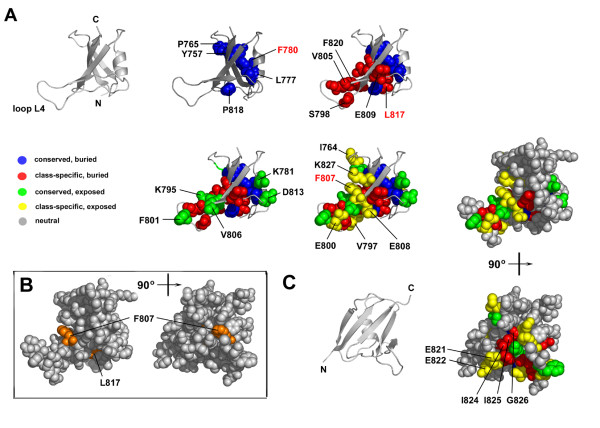
**Trace residues for partition P08 mapped onto the Axin DIX domain**. (A) The DIX domain is shown as grey ribbon representation and trace residues are shown as coloured balls: conserved and buried residues are in blue, class-specific and buried in red, conserved and surface exposed in green, class-specific and exposed in yellow. Non-trace residues are coloured grey. Residues annotated with red text have been reported in the literature (see Table 1). (B) The DIX domain shown as a ball model in grey, with residues whose substitution affect function shown in orange. (C) The Axin DIX domain rotated 90° relative to figures in (A).

**Figure 4:**
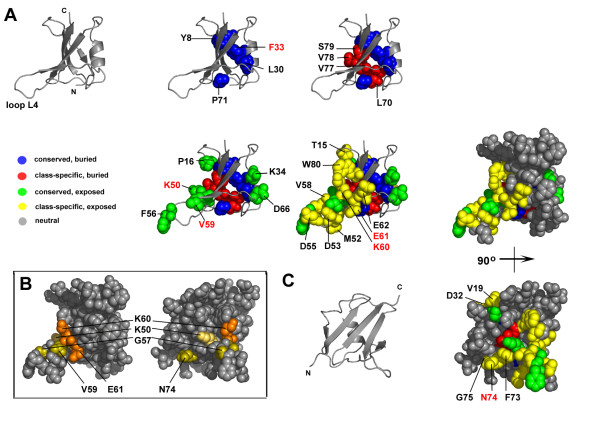
**Trace residues for partition P08 mapped onto the Dishevelled DIX domain comparative model**. (A) The DIX domain is shown as grey ribbon representation and trace residues are shown as coloured balls: annotation as in Figure 3. (B) The DIX domain shown as a ball model in grey, with residues whose substitution are known to affect function coloured: residues involved in DIX dimerization and lipid-binding are coloured orange, the residue involved in actin binding is pale brown and additionally identified residues are coloured olive (see Table 2). (C) The Dishevelled DIX domain rotated 90° relative to structures in A.

Partition P08 was chosen as the partition for which residues would be mapped onto the structures of the DIX domain. In this partition almost 40% of the sequences aligned had diverged. In later partitions, P09-10, too many trace residues were evident to make any meaningful predictions regarding their structural or functional importance. Twenty eight trace residues were identified within partition P08. These are listed in Table 1 for the Axin DIX domain and in Table 2 for the Dishevelled DIX domain. The tables list the trace category of each residue and correlate their predicted significance with known mutagenesis data, where available. Table 1 also lists the residues that form crystal contacts between the adjacent DIX domain chains in the asymmetric unit of the Axin DIX crystal structure. Of the 28 identified trace residues in Axin, 13 were buried-conserved or buried-class-specific residues and 15 were surface exposed-conserved or surface exposed-class-specific residues, whereas Dishevelled had 8 residues of the former and 20 of the latter (Figure 3 and 4). Following is a detailed analysis of each of these four trace residue categories, starting with residues that are structurally important followed by residues that are functionally important.

**Table 1:** Trace residues of the rat Axin1 DIX domain

Axin DIX trace analysis					Residues involved in DIX-DIX interactions^c^	Known single non-synonymous substitutions and their consequences		
		
DIX trace residue^a^	CB^b^	SB^b^	CE^b^	SE^b^		Mutant	Characterization	Reference
Y757	X							

I764				X	X		Required for homo-dimerization	[14]

P765	X							

T768				X				

L777	X							

Q779				X				

F780	X					F780R ^d^	Cannot form Axin homodimer	[12]

K781			X					

K795			X					

V797				X				

S798		X						

E800				X	X			[14]

F801			X					

V805		X						

V806			X					

F807				X	X	F807R ^d^	Cannot form Axin homodimer	[12,14]

E808				X	X		Required for homo-dimerization	[14]

E809		X						

D813			X					

L817		X				L817E ^d^	Cannot form Axin homodimer	[12,14]

P818	X							

F820		X						

E821				X				

E822				X				

I824		X						

I825		X						

G826		X						

K827				X	X		Required for homo-dimerization	[14]

a numbering according to rat Axin1 DIX crystal structure (1WSP)

b CB, conserved and buried; SB, class-specific and buried; CE, conserved and exposed; SE, class-specific and exposed

c based of contacts observed in the rat Axin 1 crystal structure between chain B and C of the asymmetric unit

d mouse Axin 1 mutants

**Table 2:** Trace residues of the human Dishevelled DIX domain

Dishevelled DIX trace analysis					Known single non-synonymous substitutions and their consequences		
**DIX trace residue^a^**	**CB^b^**	**SB^b^**	**CE^b^**	**SE^b^**	**Mutant**	**Characterization (genetic and/or biochemical)**	**Reference**

Y8	X						

T15				X			

P16			X				

V19				X			

L30	X						

D32				X			

F33	X				F40S (UAS-dsh^8-65^) ^c^	Loss-of-function	[29]

K34			X				

K50			X		DSH2 K58A ^d^	Unable to bind actin	[28]

M52				X			

D53				X			

D55				X			

F56			X				

V58				X			

V59			X		V66A (UAS-dsh^8-19^) ^c, e^	Loss-of-function	[29]

K60				X	DSH2 K68A ^e^	Unable to homo-dimerize; cannot interact with lipids	[11,28]

E61				X	DSH2 E69A^e^	Unable to homo-dimerize; cannot interact with lipids	[11,28]

E62				X			

D66			X				

L70		X					

P71	X						

F73				X			

N74				X	N80I (UAS-dsh^8-80^) ^c^	Loss-of-function	[29]

G75				X			

V77		X					

V78		X					

S79		X					

W80				X			

a numbering according to human Dishevelled DIX sequence

b CB, conserved and buried; SB, class-specific and buried; CE, conserved and exposed; SE, class-specific and exposed

c mutants of *Drosophila melanogaster *Dishevelled

d Dishevelled 2 actin-binding residue [28,30]

e Dishevelled 2 micelle-binding residue [30]

The absolutely conserved buried trace residues in Axin are Y757, P765, L777, F780 and P818. The equivalent positions in Dishevelled are occupied by Y8, P16, L30, F33 and P71 (Figure 3 and 4). In Dishevelled P16 is actually categorized as conserved, surface exposed, which likely reflects the fact that more of it is solvent exposed, based on the criterion stated under Methods, than in Axin. In both structures the leucine and phenylalanine are located on the α-helix and their side-chains project into the domain core. The tyrosine is located on strand β1 and the second proline on loop L5. Both form hydrophobic contacts within the core, although the hydroxyl group of the tyrosine is surface exposed. The conserved nature of these residues in all DIX homologues suggests that they are key structural determinants of the DIX fold, a hypothesis supported by mutagenesis data. A mutant of Axin in which F780 was substituted with arginine is unable to form Axin homodimers [12], whereas a mutant of Dishevelled in which the corresponding F33 is substituted with a serine exhibits a loss-of-function phenotype [29].

The class-specific buried residues, by virtue of their position, are also of structural importance but their nature is not conserved among the DIX homologues. The Axin residues L817, I824 are both part of the hydrophobic core. A mutant in which L817 is substituted with glutamic acid cannot form Axin homodimers attesting to its importance in maintaining the DIX fold for proper function [12]. The remaining Axin trace residues that fall in this category (S798, G804, V805, E809, F820, I825 and G826) are classed as buried, but all of them are partially solvent accessible. In Dishevelled the position corresponding to L817 in Axin is L70, which together with V77 and S79 form part of the Dishevelled DIX core.

In contrast to the aforementioned residues, residues that are either conserved or class-specific, but surface exposed, may by virtue of their location at the protein surface be important to protein function, i.e. Axin and Dishevelled protein-protein interactions. Most of the solvent accessible trace residues, both those that are absolutely conserved and class-specific, are clustered on one side of the domain centred on loop L4 (Figure 3 and 4), which is part of the putative Axin dimerization interface [14]. In Axin and Dishevelled, only 5 of the 16 and 4 of the 21 solvent accessible trace residues are located elsewhere, respectively. While most of these residues in Axin and Dishevelled are charged or polar, there is one notable exception. The residue F801 in Axin and the corresponding residue in Dishevelled, F56, are highly conserved and completely solvent exposed, being positioned on the tip of the turn of loop L4. Although no functional data exist for this residue, the fact that this hydrophobic residue is absolutely conserved in all 65 DIX homologues and completely solvent exposed suggests it might have a crucially important function in DIX domains. The only conserved solvent exposed residue for which functional data exists is V59 in Dishevelled, the same position in Axin being V806. Substitution of V59 in Dishevelled with alanine results in a loss-of-function Dishevelled phenotype [29]. Trace residues that fall in the conserved, surface exposed category are all likely to be required for DIX dimerization in general, since this is the only known function that the Axin, Dishevelled and DIXdc1 DIX domains share.

Surface exposed class-specific trace residues by definition occupy positions of functional importance because they are evolutionarily conserved, but their identity may vary between homologues. Such residues can be considered to be specificity determinants, residues that allow for specific interactions between domains that are otherwise similar. In the DIX domain for Axin one trace residue of this category has known functional significance (Table 1). Residue F807 on strand β4, if substituted with arginine, inhibits the formation of Axin homodimers [12]. The corresponding residue in the Dishevelled model structure is K60, which is known to be required for Dishevelled DIX homodimerization and is part of the Dishevelled lipid-binding motif [11,28,30]. Both residues are functionally important, but are not conserved in known sequences. Other residues in this category (Table 1 and 2) are likely equally important to specific DIX-DIX interactions and their substitution would likely allow the selective perturbation of these interactions (see following section).

Residues whose substitutions have no effect on DIX-DIX homotypic interactions serve as a useful negative control for trace analysis. Two substitutions in the DIX domain of mouse Axin, F759R and K796A, have no effect or only a slightly diminished ability to homodimerize [12]. Neither of these residues is well conserved in DIX domains and were therefore not identified as trace residues (residues labelled # in Figure 2). This, taken together with the fact that residues of importance to function were correctly identified in Axin and Dishevelled, validates our analysis.

### Putative functional residues

The residues that are located in the hydrophobic core of the DIX domains, if modified, could lead to structural changes that affect the ability of the domain to mediate protein-protein interactions. As mentioned above, existing mutagenesis data supports this conclusion. Substitution of the conserved F780 in Axin renders it unable to homodimerize [12] and substitution of the corresponding residue in Dishevelled, F33, exhibits a loss-of-function phenotype [29]. These observations indicate that mutagenesis experiments aimed at investigating the function of the DIX domain should be carefully designed bearing in mind that some residues will contribute to the formation of the overall fold and others to function. Trace residues in Axin and Dishevelled that are buried and either conserved or class-specific should therefore not be mutated if the desire is to investigate function without affecting the fold. Instead, the residues that are surface exposed should be selected.

Residues that are conserved and surface exposed are likely required for DIX-DIX interactions generally, since they are absolutely conserved and homotypic protein-protein interaction is the only known function all three protein families share. The trace residues likely involved in this function are K795, F801, V806 and D813 in Axin, and residues K50, F56, V59 and D66 in Dishevelled. One exception may be K781 in Axin and K34 in Dishevelled. While these residues fall into the conserved, surface-exposed trace category, their role may, exceptionally, be structural rather than functional. They do not cluster with other trace residues at the DIX dimerization interface, but are located on a surface roughly perpendicular to it. Since no other trace residues cluster there, it is unlikely to represent another functional site. Part of the lysine side chain, specifically the alpha-, beta-, gamma- and delta-carbon atoms, lie along the surface of the protein, so part of the side chain can form hydrogen bonds with underlying hydrophobic residues. These lysine residues may therefore seem absolutely conserved, because they shield these underlying residues from solvent.

Class-specific residues determine the specificity of the interaction, a conclusion that is also supported by mutagenesis data. Substitution of K60, E61 and N74 in Dishevelled and F807 in Axin all have functional consequences (Table 1 and 2). Other exposed class-specific residues have been implicated in Axin dimerization, but have not been studied experimentally. Analysis of the Axin DIX domain crystal structure implicated trace residues I764, E800, F807, E808 and K827 in the formation of inter-chain contacts in the crystal structure of the Axin DIX domain [14]. Their location is similar to that of residues that mediate homodimerization in the structurally similar PB1 domains. Given the good correlation between these data and our evolutionary sequence-structure analysis, the remaining trace residues that are so far uncharacterised should be functionally interesting. In Axin these residues include T768, Q779, V797, E821, E822, while in Dishevelled they are T15, V19, D32, M52, D53, D55, V58, E62, F73, G75 and W80.

### Residues not identified as trace residues

Several residues in the DIX domain of Dishevelled were not identified in our trace analysis, but having been substituted, have a loss-of-function phenotype in *Drosophila *[29] or have demonstrably lost their ability to activate Wnt-target gene reporters [14]. In the former case the substitutions in *Drosophila *Dishevelled that exhibit a loss-of-function are V43E, G64V and R82Q; in the latter case the substitution that leads to an inability to activate Wnt-target gene reporters is Y27D (residues in Figure 2 and Additional file 2 labelled with black *; the amino acid numbering for the first three residues is that of *Drosophila *Dishevelled, that of Y27 is for mouse Dishevelled 2 [14,29]). Some of these residues are poorly conserved in DIX-domain containing proteins (see Additional file 2) or are partially conserved within one of the three protein families and were therefore not identified in the trace analysis. Why do these substitutions have a phenotypic effect? The substitution G64V is made within loop L4, which forms part of the DIX dimerization interface. By virtue of its location on this interface close to residues mediating protein-protein interactions, it could still have an effect on Dishevelled function. The substitution V43E, at the C-terminal end of the α-helix, likely affects the domain structure, because substitution of the small buried hydrophobic valine with the larger charged glutamate may force strand β2 further form the α-helix, slightly distorting the fold. No similar structural explanation can be offered for the effect observed for substitution R82Q or Y27D. Both these residues are solvent-exposed, have no obvious structural importance and are located away from the dimerization interface. As such, they may exhibit a loss-of-function for reasons other then the loss of the DIX-DIX homotypic interaction. These two residues may perhaps be responsible for intramolecular interactions between the DIX domain and other domains within Dishevelled and Axin. Such interactions have been reported [5,6,8]. In every case, these residues may not be of general importance to DIX domain structure and function, but may be specifically important in a particular homologue.

## Conclusion

The sequence-structure based analysis presented here identifies a cluster of functionally significant residues located on loop L4 and strand β4 that contain most of the conserved residues in DIX domains. We identify residues that are structural and functional determinants of the DIX domain fold and residues that likely mediate specific homotypic interactions between Axin DIX domains and Dishevelled DIX domains. Based on the domains structural homology to PB1 domains, which are known dimerization modules, and the observed crystal contacts between chains in the asymmetric unit of the Axin DIX crystal structure, it is likely that the residues clustered on loop L4 and strand β4 constitute the dimerization interface of DIX domains in general. Existing mutagenesis data identified several residues of functional importance at this interface, supporting this conclusion. We predict that several other residues are also important to function. Investigating these should shed more light on the precise role of DIX domains as dimerization modules.

## Authors' contributions

MTE design the study, performed the modelling, analysis and drafted the manuscript. AMA critically reviewed the manuscript and provided general support and advice throughout the study. All authors read and approved the final manuscript.

## Supplementary Material

Additional file 1 **Accession numbers of sequences used in this study**. All accession numbers are those of the GenBank database. Click here for file

Additional file 2 **Structure-based multiple-sequence alignment of DIX domains from Axin, Dishevelled and DIXdc1**. Residues that are absolutely conserved have a black background, those that are conserved in a majority of sequences are shaded grey or light grey. The secondary structure assignment of the Axin 1 *Rattus norvegicus *crystal structure 1WSP is shown above the alignment. # coloured cyan are sites of non-synonymous amino acid substitution in Axin that have no effect on or only diminished ability to dimerize and * coloured cyan are unable to dimerize [12]. * coloured black are sites of non-synonymous amino acid substitution in Dishevelled that lead to a loss-of-function [29], whereas * coloured red are residues constituting the putative Dishevelled lipid-binding motif and * coloured green are required for Dishevelled actin-binding [28,30]. The amino acid number of the first and last residue of *Rattus norvegicus *Axin 1 and for *Homo sapiens *Dishevelled 1 (DSH1) are given. Each protein sequence is labelled with the protein name followed by the name of the species the sequence is from. In the case of Axin and Dishevelled, the numbers after the protein name, where present, denote orthologs. DSH, Dishevelled; XDSH, *Xenopus *Dishevelled; DIXdc1, DIX domain-containing 1. Click here for file

Additional file 3 **Dendrogram of the DIX domain containing proteins**. Partitions P01 to P10 are shown as vertical red lines. Evolutionary time-cut off increases from P01 to P10. The *Rattus norvegicus *DIX domain used to create the comparative model of the human Dishevelled 1 DIX domain are indicated with cyan coloured labels 1WSP and DIX, respectively. Yellow shaded area contains members of the Axin family, the orange shaded area members of the Dishevelled family. Click here for file

## References

[B1] Logan CY Nusse R The Wnt signaling pathway in development and disease Annu Rev Cell Dev Biol 2004 20 781 810 10.1146/annurev.cellbio.20.010403.113126 15473860

[B2] Veeman MT Axelrod JD Moon RT A second canon. Functions and mechanisms of beta-catenin-independent Wnt signaling Dev Cell 2003 5 367 377 10.1016/S1534-5807(03)00266-1 12967557

[B3] Malbon CC Wang HY Dishevelled: a mobile scaffold catalyzing development Curr Top Dev Biol 2006 72 153 166 10.1016/S0070-2153(05)72002-0 16564334

[B4] Clevers H Wnt/beta-catenin signaling in development and disease Cell 2006 127 469 480 10.1016/j.cell.2006.10.018 17081971

[B5] Fagotto F Jho E Zeng L Kurth T Joos T Kaufmann C Costantini F Domains of axin involved in protein-protein interactions, Wnt pathway inhibition, and intracellular localization J Cell Biol 1999 145 741 756 10330403 10.1083/jcb.145.4.741 PMC2133179

[B6] Kishida S Yamamoto H Hino S Ikeda S Kishida M Kikuchi A DIX domains of Dvl and axin are necessary for protein interactions and their ability to regulate beta-catenin stability Mol Cell Biol 1999 19 4414 4422 10330181 10.1128/mcb.19.6.4414 PMC104400

[B7] Salic A Lee E Mayer L Kirschner MW Control of beta-catenin stability: reconstitution of the cytoplasmic steps of the wnt pathway in Xenopus egg extracts Mol Cell 2000 5 523 532 10.1016/S1097-2765(00)80446-3 10882137

[B8] Julius MA Schelbert B Hsu W Fitzpatrick E Jho E Fagotto F Costantini F Kitajewski J Domains of axin and disheveled required for interaction and function in wnt signaling Biochem Biophys Res Commun 2000 276 1162 1169 10.1006/bbrc.2000.3607 11027605

[B9] Shiomi K Uchida H Keino-Masu K Masu M Ccd1, a novel protein with a DIX domain, is a positive regulator in the Wnt signaling during zebrafish neural patterning Curr Biol 2003 13 73 77 10.1016/S0960-9822(02)01398-2 12526749

[B10] Wong CK Luo W Deng Y Zou H Ye Z Lin SC The DIX domain protein coiled-coil-DIX1 inhibits c-Jun N-terminal kinase activation by Axin and dishevelled through distinct mechanisms J Biol Chem 2004 279 39366 39373 10.1074/jbc.M404598200 15262978

[B11] Leonard JD Ettensohn CA Analysis of dishevelled localization and function in the early sea urchin embryo Dev Biol 2007 306 50 65 17433285 10.1016/j.ydbio.2007.02.041 PMC2697034

[B12] Luo W Zou H Jin L Lin S Li Q Ye Z Rui H Lin SC Axin contains three separable domains that confer intramolecular, homodimeric, and heterodimeric interactions involved in distinct functions J Biol Chem 2005 280 5054 5060 10.1074/jbc.M412340200 15579909

[B13] Sakanaka C Williams LT Functional domains of axin. Importance of the C terminus as an oligomerization domain J Biol Chem 1999 274 14090 14093 10.1074/jbc.274.20.14090 10318824

[B14] Schwarz-Romond T Fiedler M Shibata N Butler PJ Kikuchi A Higuchi Y Bienz M The DIX domain of Dishevelled confers Wnt signaling by dynamic polymerization Nat Struct Mol Biol 2007 14 484 492 10.1038/nsmb1247 17529994

[B15] Walters KJ Goh AM Wang Q Wagner G Howley PM Ubiquitin family proteins and their relationship to the proteasome: a structural perspective Biochim Biophys Acta 2004 1695 73 87 10.1016/j.bbamcr.2004.10.005 15571810

[B16] Terasawa H Noda Y Ito T Hatanaka H Ichikawa S Ogura K Sumimoto H Inagaki F Structure and ligand recognition of the PB1 domain: a novel protein module binding to the PC motif EMBO J 2001 20 3947 3956 11483498 10.1093/emboj/20.15.3947 PMC149143

[B17] Yoshinaga S Kohjima M Ogura K Yokochi M Takeya R Ito T Sumimoto H Inagaki F The PB1 domain and the PC motif-containing region are structurally similar protein binding modules EMBO J 2003 22 4888 4897 14517229 10.1093/emboj/cdg475 PMC204459

[B18] Hirano Y Yoshinaga S Takeya R Suzuki NN Horiuchi M Kohjima M Sumimoto H Inagaki F Structure of a cell polarity regulator, a complex between atypical PKC and Par6 PB1 domains J Biol Chem 2005 280 9653 9661 10.1074/jbc.M409823200 15590654

[B19] Lichtarge O Bourne HR Cohen FE An evolutionary trace method defines binding surfaces common to protein families J Mol Biol 1996 257 342 358 10.1006/jmbi.1996.0167 8609628

[B20] Marti-Renom MA Stuart AC Fiser A Sanchez R Melo F Sali A Comparative protein structure modeling of genes and genomes Annu Rev Biophys Biomol Struct 2000 29 291 325 10.1146/annurev.biophys.29.1.291 10940251

[B21] Shi J Blundell TL Mizuguchi K FUGUE: sequence-structure homology recognition using environment-specific substitution tables and structure-dependent gap penalties J Mol Biol 2001 310 243 257 10.1006/jmbi.2001.4762 11419950

[B22] Thompson JD Higgins DG Gibson TJ CLUSTAL W: improving the sensitivity of progressive multiple sequence alignment through sequence weighting, position-specific gap penalties and weight matrix choice Nucleic Acids Res 1994 22 73 80 10.1093/nar/22.22.4673 PMC308517 7984417

[B23] Cuff JA Clamp ME Siddiqui AS Finlay M Barton GJ JPred: a consensus secondary structure prediction server Bioinformatics 1998 14 892 893 10.1093/bioinformatics/14.10.892 9927721

[B24] Laskowski RA MacArthur MW Moss DS Thornton JM PROCHECK: a program to check the stereochemical quality of protein structures J Appl Cryst 1993 26 283 291 10.1107/S0021889892009944

[B25] Holm L Sander C Protein structure comparison by alignment of distance matrices J Mol Biol 1993 233 123 138 10.1006/jmbi.1993.1489 8377180

[B26] Innis CA Shi J Blundell TL Evolutionary trace analysis of TGF-beta and related growth factors: implications for site-directed mutagenesis Protein Eng 2000 13 839 847 10.1093/protein/13.12.839 11239083

[B27] Sumimoto H Kamakura S Ito T Structure and function of the PB1 domain, a protein interaction module conserved in animals, fungi, amoeba, and plants Sci STKE 2007 re6 10.1126/stke.4012007re6 17726178

[B28] Capelluto DG Kutateladze TG Habas R Finkielstein CV He X Overduin M The DIX domain targets dishevelled to actin stress fibres and vesicular membranes Nature 2002 419 726 729 10.1038/nature01056 12384700

[B29] Penton A Wodarz A Nusse R A mutational analysis of *dishevelled *in Drosophila defines novel domains in the Dishevelled protein as well as novel suppressing alleles of *axin* Genetics 2002 161 747 762 12072470 10.1093/genetics/161.2.747 PMC1462152

[B30] Capelluto DG Overduin M Secondary structure, 1H, 13C and 15N resonance assignments and molecular interactions of the dishevelled DIX domain J Biochem Mol Biol 2005 38 243 247 15826504 10.5483/bmbrep.2005.38.2.243 PMC2613849

