# Supplementary material for: The structural and functional determinants of the Axin and Dishevelled DIX domains
Source: BMC Struct Biol. 2009 Nov 12;9:70. doi: 10.1186/1472-6807-9-70 (PMC2780430; doi:10.1186/1472-6807-9-70)
Supplement: Additional file 1 — Accession numbers of sequences used in this study. All accession numbers are those of the GenBank database. [file 1472-6807-9-70-S1.doc]

Additional file 1: GenBank accession numbers of sequences used in this study.

| Accession number | Protein | Species |
| --- | --- | --- |
| AAA74049.1 | dishevelled-1 protein | [Mus musculus] |
| NP_114008.1 | dishevelled, dsh homolog 1 | [Rattus norvegicus] |
| CAI23187.1 | dishevelled, dsh homolog 1 | [Homo sapiens] |
| NP_001012432.1 | dishevelled 1 | [Pan troglodytes] |
| XP_546713.2 | dishevelled 1 isoform 1 | [Canis familiaris] |
| XP_001252163.1 | dishevelled 1 isoform 1 | [Bos taurus] |
| XP_001362264.1 | dishevelled 1 isoform 1 | [Monodelphis domestica] |
| AAV74273.1 | dishevelled 1 | [Saimiri boliviensis] |
| XP_392577.3 | dishevelled 3 | [Apis mellifera] |
| XP_967594.1 | dishevelled 3, dsh homolog | [Tribolium castaneum] |
| NP_997813.1 | dishevelled 2, dsh homolog | [Danio rerio] |
| NP_031915.2 | dishevelled 3 | [Mus musculus] |
| XP_535822.2 | dishevelled 3 isoform 1 | [Canis familiaris] |
| NP_004414.3 | dishevelled 3 | [Homo sapiens] |
| AAT42370.1 | dishevelled | [Lytechinus variegatus] |
| XP_546582.2 | dishevelled 2 | [Canis familiaris] |
| NP_004413.1 | dishevelled 2 | [Homo sapiens] |
| XP_001374509.1 | dishevelled 3 | [Monodelphis domestica] |
| Q60838 | dishevelled homolog DVL-2 | [Mus musculus] |
| XP_001169178.1 | dishevelled 2 | [Pan troglodytes] |
| XP_001105770.1 | dishevelled 2 isoform 1 | [Macaca mulatta] |
| XP_221304.3 | dishevelled 3 | [Rattus norvegicus] |
| NP_571832.1 | dishevelled, dsh homolog 3 | [Danio rerio] |
| AAG13667.1 | dishevelled | [Hydra vulgaris] |
| AAH90218.1 | Xdsh protein | [Xenopus laevis] |
| BAD93240.1 | dishevelled | [Dugesia japonica] |
| NP_511118.2 | dishevelled isoform A | [Drosophila melanogaster] |
| EAT40012.1 | dishevelled | [Aedes aegypti] |
| BAE06823.1 | dishevelled protein homolog | [Ciona intestinalis] |
| NP_494937.1 | dsh-2 | [Caenorhabditis elegans] |
| NP_494979.3 | dsh-1 | [Caenorhabditis elegans] |
| NP_878304.1 | DIX domain containing 1 | [Danio rerio] |
| ABG25914.1 | DIX domain containing 1 isoform l | [Homo sapiens] |
| XP_001106684.1 | DIX domain containing 1 isoform 1 | [Macaca mulatta] |
| XP_001145139.1 | DIX domain containing 1 | [Pan troglodytes] |
| XP_615064.3 | DIX domain containing 1 | [Bos taurus] |
| NP_001032743.1 | DIX domain containing 1 | [Rattus norvegicus] |
| XP_417934.2 | DIX domain containing 1 | [Gallus gallus] |
| NP_835219.1 | DIX domain containing 1 | [Mus musculus] |
| XP_850708.1 | DIX domain containing 1 | [Canis familiaris] |
| XP_001381334.1 | DIX domain containing 1 | [Monodelphis domestica] |
| EAT45326.1 | axin | [Aedes aegypti] |
| XP_001120373.1 | Axin-2 | [Apis mellifera] |
| XP_781992.1 | axin | [Strongylocentrotus purpuratus] |
| XP_548025.2 | Axin 2 isoform 1 | [Canis familiaris] |
| AAT42371.1 | axin | [Lytechinus variegatus] |
| NP_733338.1 | Axin | [Drosophila melanogaster] |
| NP_056547.3 | axin2 | [Mus musculus] |
| NP_077331.1 | axin2 | [Rattus norvegicus] |
| NP_571578.2 | axin 1 | [Danio rerio] |
| NP_004646.2 | axin 2 | [Homo sapiens] |
| XP_001162941.1 | axin 2 isoform 1 | [Pan troglodytes] |
| NP_571636.1 | axin 2 | [Bos taurus] |
| XP_871703.1 | Axin-1 | [Bos taurus] |
| XP_967528.1 | axin 1 | [Tribolium castaneum] |
| AAC51624.1 | axin | [Homo sapiens] |
| NP_989822.1 | axin 2 | [Gallus gallus] |
| NP_077381.1 | axin 1 | [Rattus norvegicus] |
| XP_001365008.1 | Axin isoform 1 | [Monodelphis domestica] |
| NP_033863.1 | axin 1 | [Mus musculus] |
| NP_990275.1 | axin 1 | [Gallus gallus] |
| AAF22574.1 | axin-related protein | [Xenopus laevis] |
| XP_547220.2 | Axin-1 isoform 1 | [Canis familiaris] |
