# Supplementary figures and images for: The structural and functional determinants of the Axin and Dishevelled DIX domains
Source: BMC Struct Biol. 2009 Nov 12;9:70. doi: 10.1186/1472-6807-9-70 (PMC2780430; doi:10.1186/1472-6807-9-70)

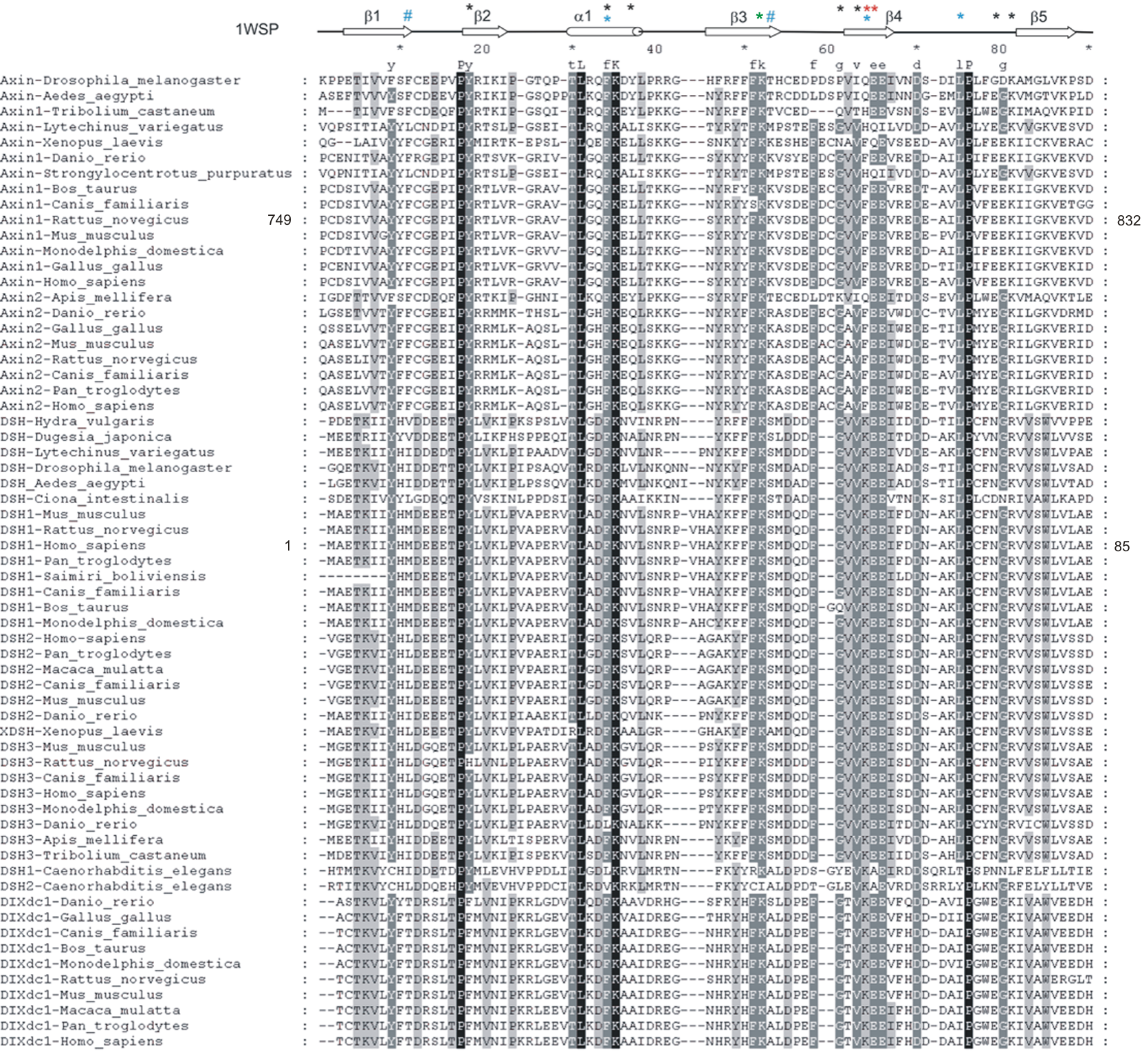

Supplement: Additional file 2 — Structure-based multiple-sequence alignment of DIX domains from Axin, Dishevelled and DIXdc1. Residues that are absolutely conserved have a black background, those that are conserved in a majority of sequences are shaded grey or light grey. The secondary structure assignment of the Axin 1 Rattus norvegicus crystal structure 1WSP is shown above the alignment. # coloured cyan are sites of non-synonymous amino acid substitution in Axin that have no effect on or only diminished ability to dimerize and * coloured cyan are unable to dimerize [12]. * coloured black are sites of non-synonymous amino acid substitution in Dishevelled that lead to a loss-of-function [29], whereas * coloured red are residues constituting the putative Dishevelled lipid-binding motif and * coloured green are required for Dishevelled actin-binding [28,30]. The amino acid number of the first and last residue of Rattus norvegicus Axin 1 and for Homo sapiens Dishevelled 1 (DSH1) are given. Each protein sequence is labelled with the protein name followed by the name of the species the sequence is from. In the case of Axin and Dishevelled, the numbers after the protein name, where present, denote orthologs. DSH, Dishevelled; XDSH, Xenopus Dishevelled; DIXdc1, DIX domain-containing 1. [file 1472-6807-9-70-S2.tiff]

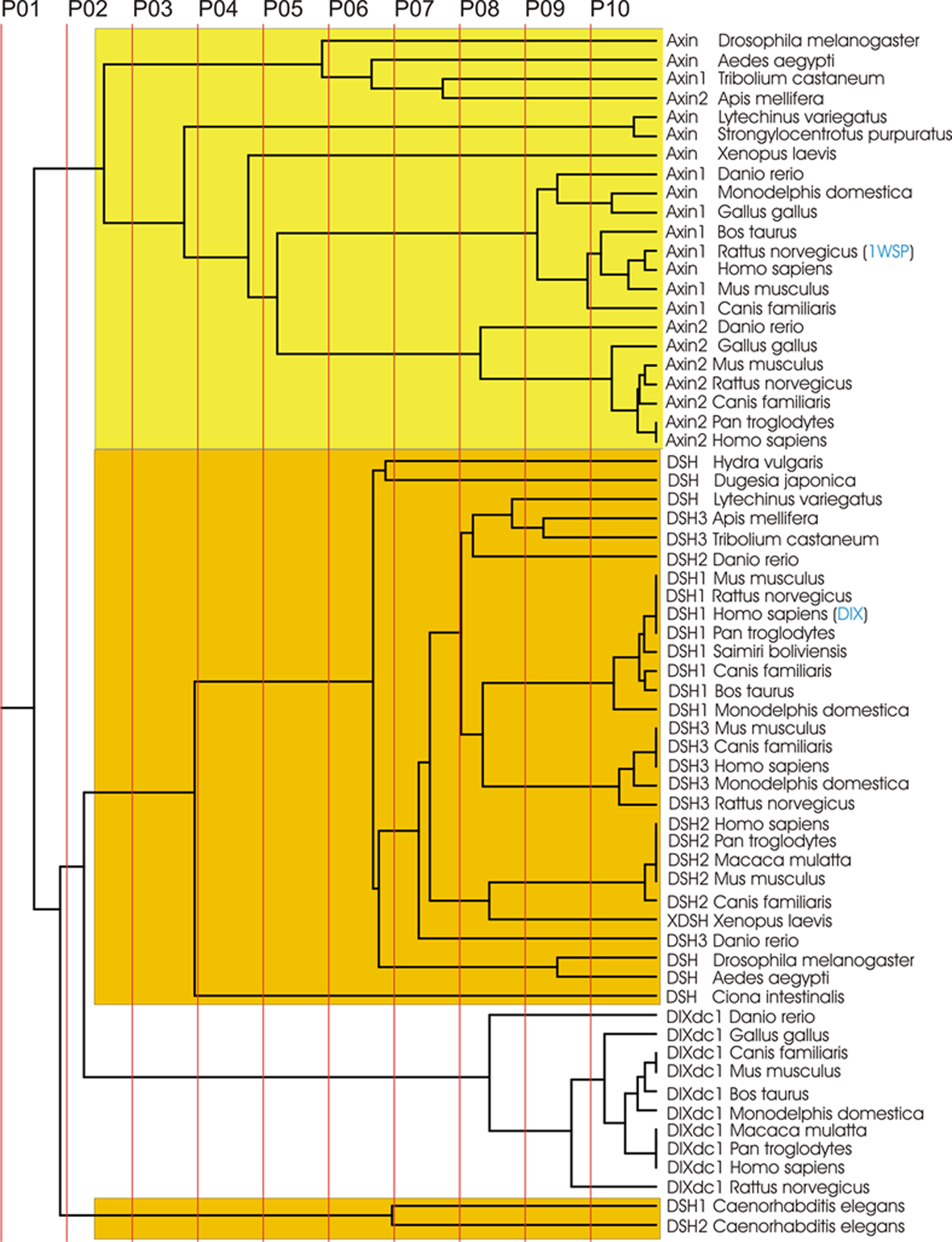

Supplement: Additional file 3 — Dendrogram of the DIX domain containing proteins. Partitions P01 to P10 are shown as vertical red lines. Evolutionary time-cut off increases from P01 to P10. The Rattus norvegicus DIX domain used to create the comparative model of the human Dishevelled 1 DIX domain are indicated with cyan coloured labels 1WSP and DIX, respectively. Yellow shaded area contains members of the Axin family, the orange shaded area members of the Dishevelled family. [file 1472-6807-9-70-S3.tiff]
